# *Morus alba*: a comprehensive phytochemical and pharmacological review

**DOI:** 10.1007/s00210-023-02434-4

**Published:** 2023-03-06

**Authors:** Gaber El-Saber Batiha, Ali Esmail Al-Snafi, Mahdi M. Thuwaini, John Oluwafemi Teibo, Hazem M. Shaheen, Ayomide Peter Akomolafe, Titilade Kehinde Ayandeyi Teibo, Hayder M. Al-kuraishy, Ali I. Al-Garbeeb, Athanasios Alexiou, Marios Papadakis

**Affiliations:** 1 Department of Pharmacology and Therapeutics, Faculty of Veterinary Medicine, Damanhour University, Damanhour, 22511 El Beheira Egypt; 2 Department of Pharmacology, College of Medicine, University of Thi-Qar, Nasiriyah, Iraq; 3 College of Medical and Healthy Techniques, Southern Technique University, Basra, Iraq; 4 Department of Biochemistry and Immunology, Ribeirão, Preto Medical School , University of São Paulo, Ribeirão Preto, São Paulo, Brazil; 5 Department of Biochemistry, University of Ibadan, Ibadan, Nigeria; 6 Department of Maternal-Infant and Public Health Nursing, College of Nursing, University of São Paulo, Ribeirão PretoRibeirão Preto, São Paulo, Brazil; 7 Department of Clinical Pharmacologyand, Therapeutic Medicine, College of Medicine , Almustansiriyah University, Baghdad, Iraq; 8 Department of Science and Engineering, Novel Global Community Educational Foundation, Hebersham, NSW 2770 Australia; 9 AFNP Med, 1030 Vienna, Austria; 10 Department of Surgery II, University Hospital Witten-Herdecke, University of Witten-Herdecke, Heusnerstrasse 40, 42283 Wuppertal, Germany

**Keywords:** *Morus alba*, Phytochemicals, Pharmacological effects, Traditional uses

## Abstract

*Morus* *alba* is a fast-growing shrub or medium-sized tree with a straight, cylindrical trunk. Medicinally, whole plants, leaves, fruits, branches, and roots have been employed. Google Scholar, PubMed, Scopus, and Web of Science were used to search for relevant material on the phytochemical components and pharmacologic and mechanism of action of the *Morus alba*. This was reviewed to assess important updates about *Morus alba*. The fruits of *Morus alba* have traditionally been used as an analgesic, anthelmintic, antibacterial, anti-rheumatic, diuretic, hypotensive, hypoglycemia, purgative, restorative, sedative tonic, and blood stimulant. Various plant parts were used as a cooling, sedating, diuretic, tonic, and astringent agent to treat nerve disorders. The plant contained tannins, steroids, phytosterols, sitosterol, glycosides, alkaloids, carbohydrates, proteins, and amino acids, as well as saponins, triterpenes, phenolics, flavonoids, benzofuran derivatives, anthocyanins, anthraquinones, glycosides, vitamins, and minerals. Previous pharmacological research identified antimicrobial, anti-inflammatory, immunological, analgesic, antipyretic, antioxidant, anti-cancer, antidiabetic, gastrointestinal, respiratory, cardiovascular, hypolipidemic, anti-obesity, dermatological, neurological, muscular, and protecting effects. This study looked at *Morus alba*’s traditional uses, chemical components, and pharmacological effects.

## Introduction

In recent decades, the field of medicinal herbs has grown rapidly. Because of their natural origins and negligible side effects, they are gaining appeal in both developing and developed countries. Plants are often secondary sources of compounds used as medications, insecticides, perfumes, colorants, biopesticides, and food additives since they are biosynthetically produced from primary metabolites. Analgesics, anti-rheumatic agents, diuretics, hypoglycemic agents, insecticides, antibacterial agents, laxatives, tonics, antihypertensive agents, and sedatives are among the many uses of Moraceae fruits.

According to chemical analysis, plants contain phytosterols, tannins, alkaloids, sitosterols, steroids, glycosides, carbohydrates, proteins, and amino acids, as well as saponins, triterpenes, phenols, flavonoids, benzofuran derivatives, anthocyanins, anthraquinones, glycosides, vitamins, and minerals (The plant list n.d.). Plants have been proven to have antibacterial, anti-inflammatory, immune, analgesic, antipyretic, antioxidant, anti-cancer, anti-diabetes, gastrointestinal, respiratory, cardiovascular, hypolipidemia, anti-obesity, dermatological, and neurological properties in previous pharmacological investigations. They have been proven to have muscle-building and protecting properties. The current study examines *Morus alba*’s traditional uses, chemical makeup, and pharmacological effects.

### Taxonomic classification

Kingdom: Plantae, subkingdom: Viridiplantae, infrakingdom: Streptophyta, superdivision: Embryophyta, division: Tracheophyta, subdivision: Spermatophytina, class: Magnoliopsida, superorder: Rosanae, order: Rosales, family: Moraceae, genus: *Morus*, species: *Morus alba* (ITIS report n.d.)*.*

### Common names

Afrikaans: gewone moerbei, witmoerbei; Arabic: Tiki, tut abiadh; Chinese: sang; English: Russian mulberry, silkworm mulberry, white mulberry; French: mûrier blanc; Indian: hipnerle, reshme chattu, kamblichedi, musukette, ambat, chinni, pippalipandu chettu, reshms chattu, shahtut, shehtun, shetur, siah; German: weißer Maulbeerbaum; Italian: Gelso bianco, moral blanco, morera blanco; Japanese: kuwa; Portuguese: amoreira-branca; Russian: šelkovica belaja; Spanish: mora, moral blanco, morera blanca; Swedish: vitt mullbär (U.S. National Plant Germplasm System n.d.).

### Distribution

Widespread in Asia (India, Palestine, Iran, China, Iraq, Japan, Afghanistan, Jordan, Korea DPR, Kazakhstan, Republic of Korea, Kyrgyzstan, Pakistan, Taiwan, Tajikistan, Turkey, Turkmenistan, Uzbekistan), Africa (Egypt, Mauritania, Mauritius, Tanzania, Tunisia, South Africa, Zambia), South America (Argentina, Brazil), Europe (former Soviet Union), and North America (USA) (U.S. National Plant Germplasm System n.d.; USDA n.d.; CABI n.d.).

## Description

*Morus alba* is a fast-growing shrub or medium-sized tree with a straight, cylindrical trunk that measures 1.8 m in circumference without buttresses. The bark is dark grayish brown in color, with longitudinal cracks and a rough surface, while the latex is white or yellowish white. The stem is lateral, scaly, and coral, with two rows of oval or nearly oval leaves, and a simple trilobal, dentate, and palm with three veins at the base. The flowers are greenish in color and have four free scale-like petals. Four stamens, pistil shape; male and loose flowers of racemes like catkins. Female flowers with long or short spikes; ovarian obstruction, 1- (2-) chamber, single ovule, two styles; fan-shaped with ovules and one ovule. A fan shape that contains the ovaries and has one ovule. Ovarian syncarpous fruit with some drupes surrounded by fleshy perianths up to 5 cm in length (Orwa et al. 2009).

## Traditional uses

The fruits have been used as analgesics, anthelmintics, antibacterial agents, anti-rheumatic agents, diuretics, antihypertensive agents, hypoglycemic agents, laxatives, tonics, and sedatives. The fruits can also be utilized as tonics for the liver and kidneys, as well as hematopoietic stimulants. Cooling, sedation, diuresis, tonicity, convergence, and neuropathy have all been treated with the roots of this plant. Twigs have been utilized as a neurotoxic and an anti-rheumatic medication. The leaves have traditionally been employed as a sweat inducer, a cooling agent, and an antipyretic (Warrier et al. 1997; Yamatake et al. 1976; Chen et al. 1995a; Chevalier 1996). Fruits were employed in traditional herbal therapy as repellants and to treat perspiration, hypertension, throat rinses, fever, and eye disorders caused by irritation of the upper respiratory tract. Root extracts have long been used as anti-inflammatory, analgesic, and protective agents in the liver and kidney systems (Katsube et al. 2006).

## Parts used medicinally

Medicinally, whole plants, leaves, fruits, branches, and roots have been employed (Plants and Morus alba. nd. Plants have sitosterol, steroids, tannins, phytosterols, glycosides, carbohydrates, proteins, alkaloids, and amino acids, as well as saponins, triterpenes, phenols, flavonoids, benzofuran derivatives, anthocyanins, anthraquinones, and glycosides, according to preliminary analysis (Nomura et al. 1983; Kusano et al. 2002; Chen et al. 2005; Imran et al. 2010). In the fruits, protein was 1.55 g/100 g dry weight, lipids were 0.48 g/100 g dry weight, crude fiber was 1.47 g/100 g dry weight, ash was 0.57 g/100 mg dry weight, total carbohydrates were 14.21 g/100 g dry weight, and moisture was 81.72 g/100 g fresh weight. Riboflavin was found to have a concentration of 3.10 mg/100 g of fresh weight, niacin 0.088 mg/100 g of fresh weight, and ascorbic acid 15.2 mg/100 g of fresh weight. Total flavanol concentrations ranged from 0.07 to 0.51 mg per gram of dry weight, while total phenol concentrations ranged from 7.7 to 11.2 mg GAE per gram of dry weight (Sánchez-Salcedo et al. 2015). Ca (0.19–0.37 g/100 g), N (1.62–2.13 g/100 g), K (1.62–2.13 g/100 g), P (0.24–0.31 g/100 g), S (0.08–0.11 g/100 g), Zn (14.89–19.58 mg/kg), B (13.78–19.48 mg/100 g), Mg (0.12–0.19 g/100 g), Na (0.01 g/100 g), Cu (4.22–6.38 mg/kg), Fe (28.2–46.74 mg/kg), Mn (12.33–19.38 mg/kg), and Ni (1.40–2.62 mg/kg) were all discovered in the fruits.

Total soluble carbohydrates were 3.4 g/100 g fresh weight, reducing sugars 1.7 g/100 g fresh weight, fructose 3.0 g/100 g fresh weight, glucose 3.1 g/100 g fresh weight, inulin 0.04 g/100 g fresh weight, nystose 0.01 g/100 g fresh weight, and fructooli 0.1 g/100 g fresh weight, all of which were found in the fruit (Rolim 2015; Al-Sayed et al. 2019). Leaves contained crude protein 13.4–24.36, crude fat 4.24–8.02%, total carbohydrate 47.27–56.42%, pectin 6.49%, lignin 0.74%, tannins 1.202%, alkaloids 20.05%, cellulose 12.84%, hemicellulose 18.99%, citric acid 32.2–105.5 mg/100 g, malic acid 43.7–72.6 mg/100 g, ascorbic acid 0.97–1.49 mg/g, oxalic acid 13 phosphorus 0.1–0.2 g/100 g, potassium 1.2–3.9 g/100 g, %, nitrogen 2.1–3.1 g/100, lithium 1.9–17.2 mg/kg, calcium 1.7–3.9 g/100 g, iron 119.3–241.8 mg/kg, sulfur 0.2–0.3 g/100 g, sodium 0.01 g/100 g, magnesium 0.5–1.4 g/100 g, molybdenum 0.8–2.3 mg/kg, manganese 35.8–90.5 mg/kg, zinc 23.9–39.5 mg/kg, copper 4.2–5.9 mg/kg, carbon 37.4–41.4 g/100 g, nickel 1.7–5.4 mg/kg, boron 253.5–825.3 mg/kg, lead 0.3–0.8 mg/kg, and titanium 5.4–10.8 mg/kg (Butt et al. 2008; Iqbal et al. 2012; Adeduntan and Oyerinde 2010; Sanchez-Salcedo et al. 2017; Khan et al. 2009; Yang et al. 2010a).

In the frost-dried powder of mulberry fruit, total phenol, total flavonoids, and anthocyanins were 23.0 mg/g gallic acid equivalent, 3.9 mg/g rutin equivalent, and 0.87 mg/g cyanidin-3-glucoside equivalent, respectively. The most common flavonols in *Morus alba* powder are rutin (0.43 mg/g), followed by morin (0.16 mg/g), quercetin (0.01 mg/g), and myricetin (0.01 mg/g) (Bae and Suh 2007). The anthocyanin concentration in the fruit alcohol extract, on the other hand, was 137–2057 mg/kg (13.70205–70 mg/100 g) (as malvidin-3-glucoside equivalent) (Chen et al. 2006). Cyanidin 3-O-(6ʺO-rhamnopyranosyl-d-galactopyranoside), cyanidin 3-O-(6ʺO-ramnopyranosyl-d-galactopyranoside), cyanidin-3-O-d-galactopyranoside, cyanidin-3-O-d-glucopyranoside, and cyanidin-3-O-d-galactopyranoside are also found. The total phenolic content of *Morus alba* ethanolic extract was 4.133 ± 0.120, 66.766 ± 0.749, and 170.200 ± 1.414 mg CAE/g, and total flavonoid content was 0.899 ± 0.014, 33.303 ± 0.059, and 52.285 ± 0.033 mg RE/g (Oliveira et al. 2016). A phytochemical study of *Morus alba* extract revealed high levels of flavonoid and cinnamic acid. HPLC fingerprinting revealed the existence of two smaller peaks related to chlorogenic acid and flavonoids, as well as two larger peaks corresponding to chlorogenic acid and flavonoids (Park et al. 2014). Mortatarins A–D flavonoids were extracted from *Morus alba* var. tatarica root bark flavonoids (Nomura et al. 1978).

From different parts of *Morus alba*, constituents such as prenylated flavonoid (moralbanone), stilbene glucoside (oxyresveratrol 3ʹ-O-beta-glucopyranoside), mulberroside A, cis-mulberroside A, oxyresveratrol, kuwanon A, B, C, E, G, J, R, S, and T, mulberroside C, and cyclomorus have been successfully isolated (Yang et al. 2011a, 2010b; Du et al. 2003; Qiu et al. 1996; Phung et al. 2012; Fitriani et al. 2019; Chen et al. 2018; Lim et al. 2014).

The ethanol extract of *Morus alba* was found to contain oxyresveratrol, two prenylflavones (cudraflavone C and cudraflavone B), and 5,7-dihydroxycoumarin-7-methyl ether (Kwon et al. 2019). *Morus alba* contains a variety of prenylated flavonoids (sanggenon C, morin, morusin kuwanon G), flavonols (isoquercitrin, quercetin, kaempferol, rutin), and alkaloids (1-deoxynojirimycin) (Memon et al. 2010). (2S)-4ʹ-hydroxy-7-methoxy-8-prenylflavan *Morus alba* leaf yielded two new flavone derivatives and twelve other well-known chemicals, including three flavones, three chalcones, benzofuran flavones, and coumarins (Natic´ MM, Dabic´ DC, Papetti A, Fotiric´ Akšic´ MM, Ognjanov V, Ljubojevic´ M and Tešic´ ŽL. 2015). Flavonoids: kaempferol 3-O-rutinoside (nicotiflorin), kaempferol 3-O-(6″-O-malonyl) glucoside, kaempferol 3-O-glucoside (astragalin), quercetin 3-O-glucoside (isoquercitrin), quercetin, quercetin 3-O-(6″-O-malonyl) glucoside, quercetin 3-O-(2″-O-malonyl)glucoside (morkotin C), quercetin 3-O-rutinoside (rutin) quercetin 3,7-di-O-glucoside, kaempferol 3,7-di-O-glucoside, and quercetin 3-O-rutinoside-7-O-glucoside (morkotin A) were all isolated from *Morus alba* fruits (Wang et al. 2014).

The total phenol content of *Morus alba* leaves ranges from 0.95 to 2.39 mg of quercetin per gram of dry extract and the total flavonoid concentration ranges from 2.64 to 7.33 mg of gallic acid per gram of dry extract. Polyphenols isolated from fruits and leaves include protocatechuic acid, galoic acid, vanyl acid, protocatechuic acid aldehyde, chlorogenic acid, syringaldehyde, syringic acid, p-hydroxybenzoic acid, ferulic acid, m-coumaric acid, kaempferol, caffeic acid, epicatechin p-coumaric acid, and rutin (Chon et al. 2009; Flaczyk et al. 2013). After separation, the fruits of *Morus alba* produced pyrrolidin-2-one, methyl 1-[2-(furan-2-yl)-2-oxoethyl] 1-[2-(furan-2-yl)-2-oxoethyl]-5-oxopyrrolidine-2-carboxylate-5-oxopyrrolidine l-pyroglutamic acid, 1-[5-(2-formlfuryl) methyl], 1-[2-(furan-2-yl)-2-oxoethyl] dihydrogen 2-hydroxypropane-1, 2, 3-tricarboxylate 2, 3-diethyl ester divaricate ester A, -2-carboxylic acid 5-O-caffeoylquinic acid methyl ester, 4-O-caffeoylquinic acid methyl ester, 3-O-caffeoylquinic acid ethyl ester, 5-O-caffeoylquinic acid ethyl ester, 3-O-caffeoylquinic acid methyl ester, l-pyroglutamic acid ethyl ester, 3-O-caffeoylquinic acid ethyl ester, 3-O-caffeoylquinic acid methyl ester, 3-O-caffeoylquinic acid, 4-O-caffeoyl quinic acid, and 4-O-caffeoylquinic acid methyl ester.

The maximum phenol concentration (117.7 2.0) was discovered in *Morus alba* methanol extract, followed by leaves (71.4 ± 2.4), twigs (49.0 ± 1.5), and fruits (11.2 ± 0.3) [mg ferulic acid equivalent (FAE) / kg dry weight]. The roots had the highest total phenol content in the fraction (166.2 ± 7.5 mg/kg dry weight for butanol and 160.8 ± 7.2 mg/kg dry weight for ethyl acetate, respectively).

Phenolic acid and flavonoids have been identified in leaf hexane extract (chlorogenic acid (3.669 ± 0.18), p-coumaric (10.334 ± 0.51), naringin (28.817 ± 1.4), and ferulic acids (0.844 ± 0.12)); in ethyl acetate extract (p-coumaric (1.005 ± 0.11) chlorogenic (2.003 ± 0.11), and ferulic acids (1.567 ± 0.11)); in butanol extract (caffeic (2.391 ± 0.11), chlorogenic (44.151 ± 2.12), p-coumaric (1.009 ± 0.11), ferulic acids (3.215 ± 0.21), and naringin (6.061 ± 0.32)); and in water extract (chlorogenic acid (14.254 ± 0.71)) (Kim et al. 2014).

Protocatechuic acid, caffeic acid, gallic acid, protocatechuic acid, vanillic acid, p-coumaric acid, and ferulic acid are examples of extracts isolated from *Morus alba*. According to Flaczyk et al., sinapinic acid has also been identified in *Morus alba* leaf extracts. Chlorogenic acid was the main phenolic component. The overall phenolic component concentration was 7.9 g per 100 g of extract, equating to 14.4 g gallic acid. The flavonol fraction contained quercetin -3-d-glucoside, rutin, and kaempferol 3-d-glucopyranoside (Kim et al. 2013).

Guibourtinidol glycosides (2R, 3S), syringaresinol-4-O-d-glucopyranoside, quercetin 7-O-dglucopyranoside, and dehydrodiconiferyl alcohol 4,9ʹdi-O-d-glucopyranoside have all been isolated from the root bark of *Morus alba* (Xie et al. 2008). Pyrrole alkaloids include morrole A, morrole B, morrole C, morrole D, morrole E, and morrole F. 2-(5-Hydroxymethyl-20,50-dioxo-20, 30, 40, 50-tetrahydro-10H-1,30-bipyrrole) carbaldehyde, 2-(5-hydroxymethyl-20,50-dioxo-20,50-dioxo-20,50-dioxo-20,50-tetrahydro-10H-1,30-bipyrrole 4-[formyl-5-(hydroxyl methyl)-1H-pyrrol-1-yl] 4-[formyl-5-(hydroxyl methyl)-1H-pyrrol-1-yl] butanoate, 4-[formyl-5-(hydroxymethyl)-1H-pyrrol-1-yl] butanoate, 4-[formyl-5-(methoxymethyl)-1H-pyrrol-1-yl] butanoic acid 2-[2-formyl-5-(methoxymethyl)-1H-pyrrole-1-yl] propanoate, 2-[2-formyl-5-(methoxy methyl)-1H-pyrrol-1-yl] 2-(50-hydroxymethyl-20-formyl pyrrole-10-yl)-3-phenyl-propionic acid lactone, 4-[formyl-5-(methoxymethyl)-1H-pyrrol-1-yl] methyl butanoic acid, methyl -3-(4-hydroxyphenyl)propanoate, (50-hydroxyl methyl-20-formylpyrrol-10-yl)-3-(4-hydroxyphenyl)—lactone—propionic acid, 2-(5-hydroxymethyl-2-formylpyrrol-1-yl)isovaleric acid lactone, 2-(5-hydroxymethyl-2-formylpyrrole-1-yl) isocaproic acid lactone, 2-(5-hydroxymethyl-2-formylpyrrole-1-yl) isocaproic acid, 3-methyl pentanoic acid lactone (hydroxymethyl) 5-hydroxy-1H-pyrrole-1-butanoic acid-1H-pyrrole-1-butanoic acid-1H-pyrrole-1-butanoi (hydroxymethyl) -1H-pyrrole-2-carboxaldehyde, lactone—2-(5-hydroxymethyl-2-formylpyrrole-1-yl)propionic acid, lactone 2-[2-formyl-5-(hydroxymethyl)-1-pyrrolyl-] 2-formyl-5-methyl pentanoic acid lactone, 2-formyl-1H-pyrrole-1-butanoic acid and 2-formyl-5-pyrrole-1-butanoic acid, and two types of 2-formyl-1H-pyrrole-1-butanoic acid and 2-formyl-5-(methoxymethyl) -1H-pyrrole-1-butanoic acid were all isolated from *Morus alba* fruits (Eruygur and Dural 2019; Sharma and Madan 1994).

1-Deoxynojirimycin was detected in *Morus alba* leaves at a concentration of 0.103–0.12%. Environmental conditions (temperature and growing duration), however, influenced the amount of chemical produced (Łochyńska and Oleszak 2011; Yao et al. 2019). *Morus alba*, had high levels of cellulose (57.4%), hemicellulose (16.3%), and lignin (13.3%) (Hunyadi et al. 2014; Chen et al. 1995b). Flavonoids such as flavonoids, alkaloids, stilbenoids, and retianone G were found in the root bark (Iqbal et al. 2012). Moriramrosid A (umbelliferone-6-dapioflanosyl (The plant list nd; Orwa et al. 2009) d glucopyranoside) and moriramrosid B (66-O (6-deoxylmannopyranosyl) d-glucopyranosyl] oxy] 2-H-1-benzopyran-1-one) were discovered in dried *Morus alba* branches. The volatile oily component of the hot water extract of *Morus alba* leaves was separated into three glycosylated volatiles (two megastigmane derivatives containing eugenol beta-d-glucoside) (Kim et al. 2012a).

In *Morus alba* leaves, N-containing sugars (fagomine, N-methyl-DNJ (N-Me-DNJ), 2-O-alpha-d-galactopyranosyl-DNJ (GAL-DNJ), 1,4-dideoxy-1,4-imino-d-arabinitol (DAB), 1,2 alpha,3 beta,4 alpha-tetrahydroxynortropan, and 1-deoxynojirimycin (DNJ)) have been identified (Yiemwattana et al. 2018).

Using ethyl acetate extract, *Morus alba* var. liquid medium from Sharon root cultures was used to isolate morushalunin, guangsangon E, chalcomoracin, kuwanon J, and kuwanon R (Yang et al. 2010b; Chen et al. 2018). *Morus alba* possesses four adducts of Diels–Alder (kuwanon J, mulberrofuran F, mulberrofuran F1, and chalcomoracin), two chalcones (isobavachalcone and morachalcone A), and three flavones (kuwanon C, norartocarpetin, and 6 geranylapigenin) (Lim et al. 2014). An aqueous methanol extract was used to isolate glycoprotein (Moran, 20 kDa) from the bark of *Morus alba* root. The protein contains more than 20% serine and cysteine, according to an analysis of its amino acid content (Jha and Srivastava 2013).

## Pharmacological effects

### Antimicrobial effects

*Morus alba* extract was tested for antibacterial activity against *P. gingivalis* and *A. actinomycetes* in inhibition zones of 10.00 ± 0.33 mm and 17.33 ± 0.58 mm, respectively. The MIC and MBC values of the *P. gingivalis* extract were 62.5 g/mL. Actinomycetemcomitans had MIC and MBC values of 250 and 500 g/mL, respectively. *P. gingivalis* LPS has been found to play a role in the generation of inflammatory cytokines. The generation of IL6 and IL8 mRNAs and proteins was significantly reduced (*p* < 0.05) when *P. gingivalis* LPS was treated with extracts at dosages of 2.5 and 5.0 g/mL (Park et al. 2003). *Morus alba* seed oil extract has been tested against *Pseudomonas aeruginosa*, *Aspergillus niger*, *Staphylococcus aureus*, *Escherichia coli*, *Saccharomyces cerevisiae*, and *Bacillus subtilis*.

The antibacterial activity of the ethanol extract was moderate, whereas the antibacterial activity of the aqueous extract was little (Gunjal et al. 2015a).

*Morus alba* ethanol extract was compared to chlorhexidine gluconate for antibacterial activity against *Porphyromonas gingivalis*, *Aggregatibacter actinomycetemcomitans*, and *Tannerella forsythia*. *Morus alba* extract is particularly susceptible to *Porphyromonas gingivalis*, which has a MIC of 1.95 mg/mL. *Porphyromonas gingivalis* and *T. forsythia* were shown to be more sensitive to chlorhexidine gluconate, with a MIC of 1.95 mg/mL (Lu et al. 2017). *Morus alb*a sol–gel and chlorhexidine sol–gel had the lowest inhibitory concentrations in actinomycete comitante, compared to *T. forsythia*, being 12 mm and 21 mm, and *P. gingivalis*, being 16 mm and 18 mm, respectively (Čulenová et al. 2020).

At 1000 mg/mL MIC, an ethyl acetate extract from a *Morus alba* branch inhibited *T. rubrum* growth. The predominant antifungal component was oxyresveratrol, which was extracted from the ethyl acetate extract. 0.500 mg/mL exhibited the minimum inhibitory concentration (MIC).

Miconazole nitrate and oxyresveratrol showed synergistic inhibitory effects when used together, as evidenced by a significant reduction in MICs for both components (Hamza et al. 2013). In vitro studies were conducted on the antiviral and antibacterial activity of phenolic compounds extracted from the bark of *Morus alba*. Plaque reduction and titer reduction assays were used to examine antiviral effects, and broth microdilution was used to test antibacterial activities. Six compounds (five prenylated compounds and one simple phenol ester) have been found to suppress the replication of herpes simplex virus 1 (HSV1) and herpes simplex virus 2 (HSV2), with IC_50_ values ranging from 0.64 to 1.93 g/mL and EC_50_ values ranging from 0.93 to 1.61 g/mL.

The antiviral activity of marveloside C against herpes simplex virus type 1 (HSV1) was poor (IC_50_: 75.4 μg/mL), but retianone G isolated from the bark of *Morus alba* was exceptional (IC_50_: 1.6 μg/mL) (Qiu et al. 1996). Extracts of *Morus alba* crude oil and seed oil show high insecticidal activity against grains of the genus *Sitophilus* (Islam et al. 2008).

Overview of the pharmacological activities of *Morus alba.*

**Table:** 

Activities	Plant part used	Activity Model	References
Anti-inflammatory activity	Twig, root	Carrageenan in mice	Chen et al., 2013; Chung et al., 2003
Antioxidant activity	Twig, leaves, fruit	Ferrous ion chelating activity, ferric reducing power	Chang et al., 2001; Yang, 2011; Yea et al., 2016; Lye, 2012
Anti-cancer activity	Leaves, root	Hepatocellular carcinoma cells, hepatoma cells	Dat, 2010; Naowaratwattana, 2010; Chan et al., 2016
Antihyperlipidemic activity	Leaves, root	High-cholesterol diet treated hyperlipidemic rats	Zeni and Dall’Molin, 2010; Jo et al., 2014
Antimicrobial activity	Leaves, root	*Pseudomonas aeruginosa*, *Escherichia coli*, *Bacillus subtilis*, *Streptococcus mutans*, *Streptococcus sanguis*, *Streptococcus sobrinus*	Omidiran et al., 2012; Park et al., 2003
Neuroprotective activity	Leaves	Foot shock-induced aggression Water maze test	Yadav and Nade, 2008; Kaewkaen et al., 2012
Antidiabetic activity	Twig, leaves, root, fruit	Alloxan-induced diabetes, brain-derived neurotropic factor, Zucker diabetic fatty rats	Liu et al., 2015; Shukla et al., 2016; Vichasilp et al., 2012; Mohammadi and Naik, 2012; Kumar, 2012; Yea et al., 2016; Sarikaphuti et al., 2013
Anti-atherosclerotic activity Leaves, Fruit Human Endothelial Cells Rynkoa et al., 2016; Harauma et al.,	Leaves, fruit Human Endothelial Cells Rynkoa et al., 2016; Harauma et al.,	Human endothelial cells	Rynkoa et al., 2016; Harauma et al., 2007; Chen et al., 2005
Anti-obesity activity Leaves, Fruit Diet-induced obese mice Obese mice Oh et al., 2009; Valacchi et al., 2014	Leaves, fruit	Diet-induced obese mice obese mice	Oh et al., 2009; Valacchi et al., 2014
Tyrosinase inhibitory activity/skin whitening activity	Twig, leaves	Melanin formation in melanA cells	Zhang et al., 2016; Lee et al., 2002
Hepatoprotective activity	Fruit, leaves	Carbon tetrachloride in rats	Hogade et al., 2010; Hsu et al., 2012
Cardioprotective activity	leaves	Cardiac markers	Madhumitha and Indhuleka, 2012

### Anti-inflammatory, analgesic, and antipyretic effects

*Morus alba* extract suppressed the production of inflammatory cytokines such as LPS-induced interleukin 6 (IL6) and tumor necrosis factor (TNF). Methanol extracts from *Morus alba* leaves and their fractions (chloroform, butanol, aqueous fraction) inhibited NO production in LPS-activated RAW264.7 macrophages at a dose of 4100 mcg/mL. *Morus alba* leaf extract and its fraction also dramatically reduced TNF alpha production (Yimam et al. 2016a). *Morus alba* root bark extract produced NO via inhibiting iNOS overexpression. It also blocked the activation of ERK1/2 by degradation and hyperphosphorylation of IB, which hindered the activation of NFkB via p65 nuclear translocation (Khunakornvichaya et al. 2016). In mice, ethanol extract had a significant inhibitory effect on acute inflammation. The presence of flavonoids and chlorogenic acid in its composition is most likely responsible (Park et al. 2014). In mice with carrageenan-induced apoemium, mulberrofuran B showed anti-inflammatory effects in vivo (El-Sayyad et al. 2011). In a rat model of osteoarthritis caused by anterior cruciate ligament amputation, the anti-nociceptive efficacy of the *Morus alba* strain extract was assessed by analyzing hindlimb weight loading and cartilage protective activities. Oral treatment with *Morus alba* strain extract (56 and 560 mg/kg) significantly reduced joint pain compared to the osteoarthritis-induced group treated with vehicle. Rats given 560 mg/kg *Morus alba* extract increased their marking scores (Sungkamanee et al. 2014).

### Immunological effects

*Morus alba* hot water extract induced rat systemic anaphylactic shock and anti-chicken gamma globulin (CGG) IgE-mediated activation of peritoneal mast cells. 48/80-induced systemic anaphylactic shock and anti-chicken gamma globulin (CGG) is used to induce IgE-mediated activation of rat peritoneal mast cells. Compound 48/80-induced cAMP decrease in RPMC was likewise significantly inhibited by the extract (Bharani et al. 2010). *Morus alba* extract (200 and 400 mg/kg, oral) had the same effect as levamisole on delayed hypersensitivity reactions. The number of white blood cells, lymphocytes, neutrophils, and eosinophils did not increase much, but the total quantity of white blood cells, lymphocytes, neutrophils, and eosinophils did. As a result, the aqueous extract of *Morus alba* stimulates the congenital or non-specific immune system in a dose-dependent manner without having an immunomodulatory effect on the adaptive immune system (Chang et al. 2015). In mouse spleen cells, polysaccharides isolated from the root cortex of *Morus alba* were evaluated for immunomodulatory activity. In the presence of mitogen, the polysaccharide elevated splenic lymphocyte proliferation while suppressing the generation of primary IgM antibodies by activated B cells (Kwon et al. 2019).

*Morus alba* fruit extract stimulated macrophages by signaling through the nuclear factor B (NFB) signaling pathway downstream of mitogen-activated protein kinase (MAP kinase) and Toll-like receptors (TLRs) (USDA, Morus alba n.d.). In RAW264.7 cells, the extract stimulated macrophage development, which resulted in phagocytic activity (Dkhil et al. 2015). According to mechanical research, oxyresveratrol suppresses the MEK/ERK signaling cascade, which inhibits CXCR4-mediated T cell mobility (Khyade 2016).

### Antioxidant effects

Hexane, ethyl acetate, butanol, and water extracts of *Morus alba* demonstrated high radical scavenging activity. The ability of plant extracts to remove free radicals has been shown to be closely related to their total phenol concentration (Kim et al. 2014). Ethanol extracts from fruits, leaves, and roots have been shown to exhibit free radical scavenging activity at IC_50_ values of 0.1469, 0.0124, and 0.0274 mg/mL, respectively (Oliveira et al. 2016). The extract significantly lowered blood urea nitrogen, plasma creatinine, and uric acid levels (Arfan et al. 2012).

*Morus alba* extract demonstrated stronger antioxidant activity and was rich in phenolic components than hydromethanol extract. Furthermore, phenol content and antioxidant activity have a strong beneficial relationship (Zhang et al. 2009). Four flavonoids derived from *Morus alba* were tested for antioxidant activity, and all four isolated substances showed DPPH and ABTS radical scavenging activity (Hunyadi et al. 2012). Two prenylflavones (cudraflavone B and cudraflavone C), oxyresveratrol, and quatethanol extract were tested for antioxidant activity. The substances oxyresveratrol and 5,7-dihydroxycoumarin-7-methyl ether, with IC_50_ values of 19.1 ± 3.6 and 3.81 ± 0.5 μmol, respectively, showed superoxide scavenging activities. The antioxidant activity of *Morus alba* ethanol extract was investigated, and it was revealed to have radical scavenging, reducing, and iron ion chelating properties. Furthermore, the phospholipids were protected from free radical damage by the ethanol extract (Singab et al. 2005). The flavonoids in the extract were in the order of quercetin > kaempferol > astragalin. In a time- and dose-dependent manner, the extract, quercetin, kaempferol, and astragalin all reduced AAPH-induced oxidative hemolysis of RBC (Wilson and Islam 2015).

### Antidiabetic effects

Ethanol extract from *Morus alba* leaves was fractionated and its antidiabetic effect was investigated in vitro. Strong fractions were tested in vivo using streptozotocin-induced diabetic rats.

One of the fractions (fraction 2) showed high antihyperglycemic efficacy due to significant changes in enzyme activity (Mahmoud et al. 2017). Aqueous extract of *Morus alba* reduced serum glucose, common cholesterol, triglycerides, LDL, and antioxidant enzyme levels in affected rats (Shams-Ardekani et al. 2013). In the liver, fruit extract increased pAMPK while lowering glucose-6-phosphatase and phosphoenolpyruvate carboxykinase. By activating AMPK and AS160 with skeletal muscle mass and suppressing gluconeogenesis in the liver, it reduced hyperglycemia and insulin sensitivity (Salama et al. 2017). In streptozotocin-induced diabetic retinopathy, the mechanism behind the protective effect of *Morus alba* leaf ethanol extract (100 mg/kg, 16 weeks) against oxidative stress, inflammation, apoptosis, and angiogenesis was also investigated. The extract was high in polyphenols and exhibited good free radical scavenging activities. Under hyperglycemic conditions, the effect of an alcoholic extract of *Morus alba* leaves on fetal fibroblast cells was tested. *Morus alba* alcoholic extract resulted in more favorable mobileular attachment and proliferation, as well as cytoprotective effects against hyperglycemia (Wang et al. 2013).

At two dose ranges (250 and 500 mg/kg), *Morus alba* was shown to significantly reduced serum glucose, amylase, TC, and renal function and increased serum HDL and TAC levels compared to the STZ diabetes group. Histological investigation of pancreatic and renal sections of diabetic rats fed *Morus alba* demonstrated a regular structure compared to the STZ diabetic group (Nazari et al. 2013). In the streptomycin-induced diabetes mouse (El-Beshbishy et al. 2006), the extracts significantly reduced glucose levels in the blood and increased antioxidant enzyme activity (SOD, CAT, GSHPX), but not only glycosylated serum proteins, but also antioxidative enzyme activity (SOD, CAT, GSHPX).

All flavonoids isolated from the root bark of *Morus alba* var. tatarica were examined for their ability to inhibit glucosidase. With IC_50_ values of 5.0 ± 0.3, 7.5 ± 0.5, and 5.9 ± 0.2 M, three drugs showed significant glucosidase inhibition (Nomura et al. 1978). Prenylated flavonoids (sanggenon C, morin, Kuwanon G, morusin), flavonols (kaempferol, rutin, quercetin, isoquercitrin), and alkaloids (1-deoxynojirimycin) have been discovered to have glucosidase inhibitory effects (Memon et al. 2010).

### Anti-cancer effects

The cytotoxic properties of morushalunin, chalcomoracin guangsangon E, and kuwanon J isolated from ethyl acetate extract in *Morus alba var* liquid media P388 cells from murine leukemia were used to investigate Shalun root cultures. Morushalunin, guangsangon E, and carcomoracin all showed significant cytotoxicity, with IC_50_ values of 0.7, 2.5, and 1.7 g/mL, respectively, while kuwanon J only showed mild cytotoxicity (IC_50_ = 5.9 g/mL) (Chen et al. 2018). The cytotoxicity of 11 flavonoids isolated from *Morus alba* leaf methanol extracts was tested using HeLa human cervical cancer, MCF7 human breast cancer, and Hep3B human hepatocellular carcinoma cells. With an IC_50_ of 0.64 M, morusin has the most potency against HeLa cells (Dabili et al. 2019). The anti-cancer effect of *Morus alba* root bark extract has been demonstrated in SW480 human colon cancer cells, which induce deodorant-dependent cell proliferation arrest and death. In SW480 cells, the extract increased ATF3 expression while decreasing cyclin D1 levels. The extract-induced ATF3 expression was dependent on ROS and GSK3β. The extract-induced downregulation of cyclin D1 was caused by ROS-dependent proteasome degradation (Qu et al. 2019).

Calu6 (human lung cancer), MCF7 (human breast adenocarcinoma), and HCT116 (human colon cancer) were among the cell lines evaluated using mulberry leaf methanol extracts (human colon cancer). On human cell lines, the antiproliferative effect of *the Morus alba* extract was varied and was linked to the concentration of the isolated extract under evaluation. At a dosage of 1 g/mL, fermentation of *the Morus alba* leaves increased the antiproliferative action of methanol extract on human gastric cancer cell line (SNU601) (Kim et al. 2014). Bcl2 levels were lowered and Bax levels were elevated in A172GBM cells treated with *Morus alba* leaf flavonoid extract, doxorubicin, or a combination of flavonoid extract and doxorubicin. The percentage of cells that went into apoptosis was likewise much higher in the treated cells (Kikuchi et al. 2010).

The cytotoxicity of *Morus alba* root extracts was studied against neuroblastoma cell lines (B103) and normal cells (Rat2). Extract treatment results in increased reactive oxygen species (ROS), depolarization of mitochondrial membrane potentials in B103 cells, DNA damage, and death. A decrease in cell proliferation and transcription was also found to be associated with apoptosis, according to studies of pAkt expression. The enhanced activity of Bax and the cleaved caspase 3 further demonstrated this (Nade et al. 2013).

*Morus alba* extract reduced hepatocellular cancer, dysplastic nodules, lipid peroxidation, protein carbonylation, and DNA degradation (Pirvulescu et al. 2011). In a dose-dependent manner, cyanidin-3-lucinoside and cyanidin-3-glucoside reduced the migration and infiltration of highly metastatic A549 human lung cancer cells (both derived from *Morus alba*). The results show that cyanidin-3-glucoside and cyanidin-3-lutinoside therapy suppress matrix metalloproteinase 2 tissues by dose-dependently reducing the expression of MMP2 and urokinase plasminogen activator (uPA) and elevated levels of drug (TIMP2) and plasminogen activator inhibitors (PAI). In addition, cyanidin-3-lucinoside and cyanidin-3-glucoside inhibited the activation of cJun and NFkappaB (Du et al. 2008).

### Cardiovascular effects

*Morus alba* reduced isoproterenol-induced myocardial damage, resulting in smaller regions of myocarditis and myocardial necrosis in treated rats, as well as lower levels of cardiac markers. In a myosin-induced myocarditis model, it also retained cardiac tissue without substantial infiltration of inflammatory cytokines and fibrous tissue, correcting systolic and diastolic dysfunction of the myocardium (Naowaboot et al. 2009a; Yang et al. 2014b).

*Morus alba* significantly suppressed expression of periactin and fractalkine, as well as intracellular ROS levels, NADPH activation, and increased monocyte attachment to human endothelial cells (Yang et al. 2012). *Morus alba* reduced abnormally mean arterial pressure and heart rate, high systolic, and diastolic blood pressure in experimental mice. The impaired reactivity of blood vessels (including diminished dilatation and increased constriction) was restored to the normal levels after long-term treatment of mulberry leaves. After long-term therapy with *Morus alba* leaves, the impaired vascular reactivity (including decreased dilation and increased contraction) returned to normal levels. In vascular smooth muscle cells, relaxation was mediated by voltage-gated and receptor-dependent Ca^2+^ channel blockage, while contraction was mediated by activation of the sarcoplasmic reticulum ryanodine receptor (Naowaboot et al. 2009b). The plant extract caused a short decrease in blood pressure and pulse rate lasting less than 3 min (Lee et al. 2011). In a mouse model of arterial hypertension, *Morus alba* extract had an antihypertensive effect. Endothelial vascular relaxation was induced by the extract via a nitric oxide-dependent route. Increased phosphorylation of endothelial nitric oxide synthase (eNOS) was discovered through molecular research. It also had a positive impact on the vascular system by activating two key proteins that act as stress receptors (Hong et al. 2011).

*Morus alba* extract or 4U/kg insulin effectively improved vascular responsiveness in diabetic rats. After treatment with *Morus alba* extract, malondialdehyde levels in the liver, kidneys, heart, and aorta were all significantly reduced (Sharma et al. 2010). In atherosclerotically fed rats, chronic treatment with low-dose (100 mg/kg/day) or high-dose (200 mg/kg/kg) extracts reduces hypertension and suppresses acetylcholine-induced vascular ring relaxation. Treatment with *Morus alba* leaves restored circulating endothelial dysfunction markers (soluble vascular cell adhesion molecule 1, fibrinogen and nitric oxide) to normal levels (Yang et al. 2011b). *Morus alba* leaves were also efficient against atherosclerotic plaques that had already formed. The amount of plaque was significantly reduced in animal trials after long-term therapy with *Morus alba* (Alvin et al. 2011).

Using isolated rat thoracic aorta, the vasoprotective effect of *Morus alba* root bark extract was investigated. Via an endothelium-dependent mechanism, the extract caused concentration-dependent vasorelaxation. After the endothelium was removed, vascular relaxation in response to the extract was significantly reduced. The extract also lowered the contractile response to phenylephrine by a significant amount (Woo et al. 2017).

### Dermatological effects

The effect and mechanism of action of *Morus alba* extract on B[a]P-induced cytotoxicity in human keratinocytes were investigated. After pretreatment with *Morus alba* extract, B[a]P induced (AhR) nuclear translocation and aryl hydrocarbon receptor activation were reduced. The extract reduced DNA damage and restored the delay in the S phase of cell cycle by inhibiting the development of DNA adducts derived from B[a]P in a dose-dependent method. In B16F10 melanoma cells, the *Morus alba* tree methanol extract significantly lowers intracellular tyrosinase and melanin levels, making it one of the top five most effective extracts. Among identified compounds, 2,4,3ʹ-trihydroxydihydro stilbene was discovered to be a new powerful tyrosinase inhibitor (Oh et al. 2009). *Morus alba* leaves have been shown to suppress the development of melanin, and the active components in *Morus alba* are now being researched.

Methanol extract from UV-irradiated *Morus alba* (UVCIML) had a greater tyrosinase inhibitory action than unirradiated *Morus alba*.

## Toxicity and adverse effects

In mice, the LD_50_ of *Morus alba* leaf extract was 2 g/kg (Nomura et al. 1983). The oral toxicity of *Morus alba* aqueous extract was studied in rats for 28 days (0, 1, 2, 4 g/kg body weight/day). There have been no recorded treatment-related deaths or adverse events, and no target organ has been identified (based on clinical observation, weight/weight increase, diet, ocular examination, clinical pathology, gross pathology, organ weight, or histopathology). The greatest dose (4 g/kg body weight/day) had no effect on male or female rats (Food and Agriculture Organization 2002). The oral toxicity of *Morus alba* ethanol extract at a dose of 300 mg/kg is not fatal and does not cause histological abnormalities in the liver, spleen, or kidneys of mice, only causing a decrease in white blood cell count (Prasad et al. 1995). In vivo genotoxicity and acute toxicity of ethanol extracts from *Morus alba* leaves were examined in mice at dosages of 300 and 2000 mg/kg body weight for 14 days on ip. There were no fatalities or behavioral problems in the treated mice in the toxicity investigation when compared to the dosage controls. Biochemical, hematological, and histological analyses, on the other hand, revealed that intraperitoneal injection generated pathological alterations. The extract did not cause genotoxicity when taken orally (Park et al. 2014). For 14 days, mice were tested for acute toxicity of *Morus alba* ethanol extract at 300 and 2000 mg/kg BW ip. Toxicity was determined by counting the number of micronucleated polychromic red blood cells in the blood of mice administered 75, 150, and 300 mg/kg BW orally. The mice did not die or change behavior when compared to any dose of control. However, after intraperitoneal injection, the extract causes hematological, biochemical, and histological changes. At all doses tested by negative control, oral administration of the extract did not cause genotoxicity or significant leukocyte migration (58, 65, 6% inhibition) (Park et al. 2014). The acute and chronic toxicity of *Morus alba* root bark extract was investigated in mice. The extract was administered subcutaneously at a dose of 50–200 mg/kg/day for 3 weeks and 3 months in subacute and chronic toxicity examination. The extract has no adverse effects on the animal (Lee et al. 2011). The toxicity of UP1306, a standardized mixture of *Morus alba* root bark extract and acacia catechu utilized as a commercial dietary supplement for joint care (500, 1000, 2000 mg/kg, 28 days orally), was investigated. There was no evidence of morbidity or mortality. In terms of body weight, food intake, hematological, clinical chemistry, organ weight, gross pathology, and histology, there were no significant changes between the groups (Prasad and Reddy 1991).

*Morus alba* leaf ingestion by humans and animals around the world has a long history, implying that the leaves and extracts are largely harmless. *Morus alba* leaves can be fed to cattle, sheep, goats, pigs, chickens, and rabbits (including pregnant animals) at up to 75% of their diet without causing health problems [191–194].

## Conclusion

This review offered a comprehensive assessment of *Morus alba*’s phytochemical and pharmacological properties, as well as its safety and efficacy, as a prospective herbal medicine. The plant contained tannins, steroids, phytosterols, sitosterol, glycosides, alkaloids, carbohydrates, proteins, and amino acids, as well as saponins, triterpenes, phenolics, flavonoids, benzofuran derivatives, anthocyanins, anthraquinones, glycosides, vitamins, and minerals. Pharmacological research identified antimicrobial, anti-inflammatory, immunological, analgesic, antipyretic, antioxidant, anti-cancer, antidiabetic, gastrointestinal, respiratory, cardiovascular, hypolipidemic, anti-obesity, dermatological, neurological, muscular, and protecting effects.

## Data Availability

Data sharing is not applicable to this article as no datasets were generated or analyzed during the current study.
